# Safety Concerns of Nasal Corticosteroids Usage in Patients With Allergic Rhinitis

**DOI:** 10.7759/cureus.11651

**Published:** 2020-11-23

**Authors:** Talal A Almutairi, Abdulaziz A Aldayel, Abdulrahman S Aldayel, Fahad Alotaibi, Hamad A Alhussain

**Affiliations:** 1 Medicine, Imam Mohammad Ibn Saud Islamic University, Riyadh, SAU; 2 Otolaryngology, Imam Mohammad Ibn Saud Islamic University, Riyadh, SAU

**Keywords:** intranasal corticosteroids, allergic rhinitis, intranasal steroids safety

## Abstract

Background

Intranasal corticosteroids (INCSs) are the first-line treatment for patients with moderate to severe conditions of allergic rhinitis (AR) as per current guidelines. However, patients' knowledge and practice towards the safety of such medications remains ambiguous. Therefore, this study was undertaken to identify the awareness of and knowledge about the safety of nasal corticosteroid usage in patients with allergic rhinitis as well as their adherence to taking the medication.

Materials and methods

We conducted a cross-sectional study from June to September 2020 at Imam Mohammad Ibn Saud Islamic University Medical Center, Riyadh, Kingdom of Saudi Arabia. Data were collected through questionnaire-based surveys, and a total of 375 patients were enrolled in the study. The eligibility criteria included all adult patients diagnosed with allergic rhinitis.

Results

Most of the patients had used intranasal corticosteroids. However, only two-fifths of patients stated these medications were effective and only 27% thought they were safe to use. More than half of the patients expressed concerns about using intranasal corticosteroids; however, there was no difference among the patients when asked if their concerns made them discontinue their medication. The majority of patients (73.3%) did not receive appropriate advice on how to use intranasal corticosteroids, but most were compliant with the therapy regardless of their uncertainty about the medication’s safety (71.5%). Most patients reported a benefit of using intranasal corticosteroids (71.5%). Half of the patients (53.9%) reported being aware of a special technique for how to use a nasal spray, but the nonsmokers were more knowledgeable about the techniques than the smokers (p = 0.007).

Conclusion

The patients' knowledge about, adherence to, and perceptions of intranasal corticosteroid use were found to be suboptimal. Patients’ age, gender, socioeconomic status, education level, and smoking status were recognized as potential barriers to a positive perception of and adherence to the treatment plan. Corrective measures are needed to ensure better health outcomes.

## Introduction

The term allergic disease refers to a widely recognized group of medical conditions caused by the immune system’s exaggerated response to a typically harmless environmental antigen [1]. Allergic rhinitis (AR) is considered to be a common chronic respiratory disease in the general population, and it is one of the most common chronic diseases of childhood, representing a major public health concern in many countries worldwide [2-5]. In terms of prevalence, AR is estimated to affect from 10% to 35% of the population; however, these percentages could be inaccurate because some patients do not consider AR to be a disease [6,7]. In Saudi Arabia, a study conducted on 807 residents of the city of Al-Ahsa revealed that 76% of the study sample had been diagnosed with AR and had symptoms suggestive of AR [8]. Another study concerning the prevalence of AR among children in the Jazan Region of Saudi Arabia reported that out of 1052 participants, 25.7% suffered from AR symptoms while only 24.5% had been diagnosed with the disease [9].

AR is a non-contagious inflammatory disease; it is caused by nasal hypersensitivity where immunoglobulin E is induced after a single allergen encounter, which results in an allergic reaction triggering inflammation [6,10]. The inflammatory process caused by AR is similar to that found in other atopic inflammatory disorders, such as asthma, allergic conjunctivitis, and rhinosinusitis; a high asthma prevalence has been found in patients with severe cases of AR [11,12]. The immediate allergic reaction of AR triggers nasal symptoms, such as sneezing, itching, rhinorrhea, and nasal congestion, as well as ocular symptoms, including tearing, itching, and redness [10,13]. Environmental factors are believed to have a significant effect on the development of AR symptoms [14]. Another local study in Saudi Arabia conducted on 3,458 participants reported that dust was the main trigger for AR episodes [15]. It is important to note that Saudi Arabia is one of the countries known to have frequent sandstorms throughout the year [16].

Regarding the management approach of AR, avoidance of allergen exposure has been considered to be a conservative treatment option; however intranasal corticosteroids (INCSs) have been established as the pharmacological treatment of choice for managing AR [17-20]. The use of topical steroids has been shown to control symptoms and improve quality of life and sleep, with minimal side effects. The most common side effects of INCSs result from the local irritation and include dryness, a burning sensation, blood-tinged secretions, and epistaxis [17]. Although INCSs have been proven to be the best medication for alleviating AR symptoms, some patients tend to use alternative non-pharmacological treatments, but their effectiveness is subjected to multiple factors that cannot be either proven or disproven [15]. Moreover, the management of AR is readily available, and nasal glucocorticoid is the best single medication for patients with persistent or moderate-to-severe symptoms, and its effectiveness has been previously studied [1,17].

However, patients’ noncompliance and concerns regarding the side effects of medications used to treat AR have been known to be the most common reason for treatment failure [21]. Neglected AR imposes a significant emotional, social, and financial burden on patients and subsequently impairs their social life, work productivity, academic performance, and quality of sleep [22,23]. Previous literature has emphasized the lack of knowledge about INCSs and the necessity of patient education regarding safety concerns, the correct way to use these medications, and the assurance of their tolerance and satisfaction; this will eventually result in better patient compliance and health care outcomes [6,7,20,21]. No previous studies have been conducted in the region to address this issue despite the high-risk factors associated with AR and the availability of treatment. Therefore, the present study was conducted to assess the awareness of and knowledge about the safety of INCS usage in patients with AR, and to determine their practice and adherence to taking the medication.

## Materials and methods

This study adopted an observational, cross-sectional design to investigate the awareness of and knowledge about the safety of INCSs usage in patients with AR, and to determine their practice and adherence to taking the medication. The data were collected between June and September 2020. The sample population was selected via convenience sampling; all the patients diagnosed with AR at Imam Mohammad Ibn Saud Islamic University (IMSIU) Medical Center, Riyadh, Kingdom of Saudi Arabia, were eligible and asked to participate in an online questionnaire. The participants were selected from the hospital database and encouraged to complete the questionnaire at their convenience during the time period of the study. Patients who were unwilling to participate were excluded from this study.

We collected the data through questionnaire-based surveys using a custom structured questionnaire specifically developed by the research team for this study with the aid of previous reports in the literature [7,21]. The questionnaire was reviewed and modified by a panel of experts. The questionnaire was first written in English, then translated into a local language (Arabic). A pilot study with 30 patients was conducted before the data were collected to ensure the clarity and consistency of the questions. The questionnaire was subdivided into two main sections. The first section was dedicated to gathering demographic information, including age, sex, nationality, residency, occupation, educational level, smoking status, and average household income. The second section included questions to assess the patients’ perceptions of the safety of intranasal steroids using multiple-choice questions, yes or no answers, and a 5-point Likert scale question.

Statistical Package for the Social Sciences (SPSS) version 24 (IBM, USA) was used for the analyses. The results of the descriptive analyses are presented using frequency distributions, percentages, and bar charts. Cross tabulation and the chi-square test of independence analyses were used at the bivariate level of analysis to examine the association between the participants’ demographic variables and their level of knowledge as well as their adherence to taking INCSs and their perceptions of these drugs. At the multivariate level of analysis, multinomial logistics regression was used to determine the adjusted odds ratios (ORs) of the significant variables regarding the participants’ level of knowledge about, perception of, and adherence to taking the medication. Hypothesis testing was performed at a 5% level of significance.

All the patients were informed of the study’s purpose and were requested to provide informed consent before proceeding to the questionnaire. Their participation was voluntary, and their anonymity and confidentiality were ensured.

## Results

Sociodemographic data

A total of 375 patients participated in the study. Of those, 60.5% were male (n = 227) and 39.5% were female (n = 148). The average age of the included patients was 34.4 ± 10.84; and 37.3% (n = 140) of the sample consisted of individuals ranging in age between 18 and 28. Moreover, 85.1% of the sample are Saudis (n = 319), 96.3% live in the city of Riyadh (n = 361), 64.5% are employed (n = 242), and 65.9% have a bachelor’s degree (n = 247). Furthermore, 89.6% (n = 336) of the participants are nonsmokers. The sociodemographic characteristics of the participants are presented in Table 1.

**Table 1:** Sociodemographic characteristics of the study population

Characteristics	Value n (%)
Age, mean ± SD	34.4 ± 10.84
18–28	140 (37.3)
29–38	118 (31.5)
39–58	105 (28.0)
59-Older	12 (3.2)
Gender	
Female	148 (39.5)
Male	227 (60.5)
Nationality	
Non-Saudi	56 (14.9)
Saudi	319 (85.1)
Residency	
Within Riyadh	361 (96.3)
Outside Riyadh	14 (3.7)
Occupation	
Employed	242 (64.5)
Unemployed	43 (11.5)
Student	90 (24.0)
Education level	
No formal education	1 (0.3)
Primary school	1 (0.3)
Intermediate school	4 (1.1)
Secondary school	25 (6.7)
University or college	247 (65.9)
Postgraduate	97 (25.9)
Socio-economic status	
Low	211 (56.3)
Middle	125 (33.3)
High	39 (10.4)
Smokers	
Yes	39 (10.4)
No	336 (89.6)

Assessment of the participants’ knowledge about and adherence to using intranasal steroid sprays

Of the 375 participants, 41.9% (n = 157) thought that intranasal steroids are effective and 72.5% (n = 272) would use them if they were prescribed. Furthermore, only 27.8% of the participants thought that nasal steroids are safe. However, 68.3% (n = 256) of the participants had previously used intranasal steroids and 54.7% (n = 205) expressed their concerns about using these drugs. This concern subsequently led to discontinuation of the medication by 37.6% (n = 141) of the studied sample. Moreover, 60.5% (n = 227) of the participants reported that their physician did not explain the side effects associated with these steroids. Nonetheless, 75.2% (n = 282) of participants have benefited from using nasal steroids, and 71.5% (n = 268) have been compliant in using nasal steroids as prescribed. Further details about the participants’ knowledge about and adherence to using intranasal steroids are presented in Table 2. 

**Table 2:** Knowledge, attitude, and adherence to intranasal steroids use

	Value n (%)
Knowledge of intranasal steroids spray	
I have heard of them, but I do not know much about them.	141 (37.6)
I have never heard of them before.	59 (15.7)
They are unsafe drugs.	18 (4.8)
They are effective/important drugs.	157 (41.9)
Usage of nasal steroids if prescribed	
I would use them if they were prescribed.	272 (72.5)
First, I would check the drug prospectus.	51 (13.6)
I would double-check the necessity for steroids with another physician.	35 (9.3)
I would not use them.	17 (4.5)
Would you do the same for every other prescribed drug?	
I would use them if they were prescribed.	227 (60.5)
First, I would check the drug prospectus.	91 (24.3)
I would double-check the necessity for steroids with another physician.	39 (10.4)
I would not use them.	18 (4.8)
Reason for discontinuing nasal steroid spray	
I am concerned about the long-term side effects.	99 (26.4)
It did not improve my symptoms after one week.	29 (7.7)
I do not think a nasal steroid is helpful.	26 (6.9)
Other reasons	127 (33.9)
Nasal steroids are safe	
Strongly agree	25 (6.7)
Agree	79 (21.1)
Neutral	208 (55.5)
Disagree	50 (13.3)
Strongly disagree	13 (3.5)
Usage of intranasal steroids spray before	
Yes	256 (68.3)
No	119 (31.7)
Benefit from using nasal steroids	
Yes	282 (75.2)
No	93 (24.8)
Concerns about using nasal steroids	
Yes	205 (54.7)
No	170 (45.3)
Have your concerns stopped you from taking nasal steroids?	
Yes	141 (50.2)
No	140 (49.8)
Compliance in using nasal steroids as prescribed	
Yes	268 (71.5)
No	107 (28.5)
Discontinuation of using nasal steroids after prescribed	
Yes	218 (58.1)
No	157 (41.9)
Awareness of a special technique for how to use a nasal spray	
Yes	202 (53.9)
No	173 (46.1)
Did your physician explain how to use nasal steroids?	
Yes	275 (73.3)
No	100 (26.7)
Did your physician explain the side effects of nasal steroids?	
Yes	148 (39.5)
No	227 (60.5)

Participants’ knowledge of the side effects of intranasal steroids

Most of the participants did not think that high blood pressure, high blood sugar, high intraocular pressure, obesity, and osteoporosis are side effects of using steroids. However, 24.2% of them recognized a burning sensation inside the nose as a possible consequence of using steroid sprays (Table 3). 

**Table 3:** Participants’ knowledge about the side effects of intranasal steroids use

	Value n (%)
High blood pressure is a result of intranasal steroid use	
Yes	43 (11.5)
No	332 (88.5)
High blood sugar is a result of intranasal steroid use	
Yes	30 (8)
No	345 (92)
High intraocular pressure is a result of intranasal steroid use	
Yes	48 (12.8)
No	327 (87.1)
Nasal bleeding is a result of intranasal steroid use	
Yes	69(18.4)
No	306(81.6)
Burning sensation inside the nose is a result of intranasal steroid use	
Yes	91(24.2)
No	284(75.8)
Osteoporosis is a result of intranasal steroid use	
Yes	29(7.7)
No	346(92.3)
Obesity is a result of intranasal steroid use	
Yes	45 (12)
No	330 (88)

Association between sociodemographic characteristics with knowledge about and adherence to using nasal steroids

Table 4 presents the cross-tabulation and chi-squared test of independence results of the association between the demographic variables and the participants’ knowledge about and adherence to using nasal steroids. There is a significant association between age and being concerned about using nasal steroids (p = .025) and discontinuing their use due to those concerns (p < .001). Additionally, the physicians' explanations of the side effects (p < .001) and the participants’ awareness of the special technique for using intranasal steroid sprays (p = .009) were significantly related to age. 

**Table 4:** Sociodemographic association with perceptions of and adherence to the use of nasal steroid sprays

Characteristics				Total	p Value
Age	Concerns about using nasal steroids				.025
	No	Yes			
18–28	53	87		140	
29–38	51	67		118	
39–58	60	45		105	
59 and older	6	6		12	
Total	170	205		375	
	Have your concerns stopped you from taking nasal steroids?				< .001
	No		Yes		
18–28	43		70	113	
29–38	47		47	94	
39–58	48		20	68	
59 and older	2		4	6	
Total	140		141	281	
	Did your physician explain the side effects of nasal steroids?				< .001
	No	Yes			
18–8	65	75		140	
29–38	82	36		118	
39–58	74	31		105	
59 and older	6	6		12	
Total	227	148		375	
	Awareness of a special technique for how to use a nasal spray				.009
	No	Yes			
18–28	53	87		140	
29–38	69	49		118	
39–58	45	60		105	
59 and older	6	6		12	
Total	173	202		375	
Gender	Previous use of an intranasal steroid spray				0.04
	No	Yes			
Female	56	92		148	
Male	63	164		227	
Total	119	256		375	
	Discontinuation of using nasal steroids after prescribed				0.003
	No	Yes			
Female	76	72		148	
Male	81	146		227	
Total	157	218		375	
Socioeconomic status	Previous use of an intranasal steroid spray				0.043
	No	Yes			
Low	67	144		211	
Middle	46	79		125	
High	6	33		39	
Total	119	256		375	
Education level	Concerns about using nasal steroids				0.012
	No	Yes			
No formal education	0	1		1	
Primary school	1	0		1	
Intermediate school	4	0		4	
Secondary school	10	15		25	
University or college	100	147		247	
Postgraduate	55	42		97	
Total	170	205		375	
	Did your physician explain the side effects of nasal steroids?				0.03
	No	Yes			
No formal education	0	1		1	
Primary school	0	1		1	
Intermediate school	0	4		4	
Secondary school	4	21		25	
University or college	55	192		247	
Postgraduate	41	56		97	
Total	100	275		375	
	Adherence to using nasal steroids as prescribed				0.043
	No	Yes			
No formal education	0	1		1	
Primary school	0	1		1	
Intermediate school	3	1		4	
Secondary school	6	19		25	
University or college	61	186		247	
Postgraduate	37	60		97	
Total	107	268		375	
	Awareness of a special technique for how to use nasal spray				0.030
	No	Yes			
No formal education	1	0		1	
Primary school	1	0		1	
Intermediate school	1	3		4	
Secondary school	7	18		25	
University or college	107	140		247	
Postgraduate	56	41		97	
Total	173	202		375	
Currently Smoking	Adherence to using nasal steroids as prescribed				0.028
	No	Yes			
No	90	246		336	
Yes	17	22		39	
Total	107	268		375	
	Benefit from using nasal steroids				0.013
	No	Yes			
No	77	259		336	
Yes	16	23		39	
Total	93	282		375	
	Awareness of a special technique for how to use nasal spray				0.007
	No	Yes			
No	147	189		336	
Yes	26	13		39	
Total	173	202		375	

Another significant association was noted between gender and the use of an intranasal steroid spray (p = .04). Moreover, more males than females stopped using steroids after they were prescribed (p = .003). Furthermore, the participants’ socioeconomic status was significantly related to the use of an intranasal steroid spray (p = .043).

The result of the chi-square analysis showed a significant relationship between education level and being concerned about the use of nasal steroids (p = .012) and the physicians’ explanation of the side effects of nasal steroids (p = .03). Furthermore, adherence to using nasal steroids as prescribed (p = .043) and awareness of the special techniques for doing so (p = .030) were significantly related to education level.

A significant relationship was noted between smoking status and awareness of the special technique for how to use a nasal spray. Nonsmokers were more aware of how to use a nasal spray than those who smoke (p = .007). Moreover, adherence to using a nasal spray (p = .028) and the benefits derived from doing so (p = .013) were significantly associated with smoking status.

Predictors of the participants’ knowledge about and adherence to using nasal steroids

Table 5 shows the adjusted ORs of the significant variables. It was found that participants with a low and middle socioeconomic status were less likely to ever use intranasal steroids (OR = 0.360, p = .033) and (OR = 0.313, p = .017), respectively. Nonsmokers were more likely to ever use intranasal steroids, and more likely to be aware that there is a special technique for how to use a nasal spray (OR = 2.444, p = .013) and (OR = 2.611, p = .010), respectively. 

**Table 5:** Adjusted ORs and 95%CI of predictors for participants’ perceptions of and adherence to using nasal steroids ORs: odds ratios; 95%Cl: 95% confidence intervals

Variables		p Value	OR	Lower 95% CI	Upper 95% CI
Previous use of an intranasal steroid spray					
Socioeconomic status	Low	.033	.360	.140	.921
	Middle	.017	.313	.120	.812
	High				
Smokers	No	.013	2.444	1.207	4.951
	Yes				
Awareness of a special technique for how to use nasal spray					
Smokers	No	.010	2.611	1.262	5.399
	Yes				

Benefits and side effects of using intranasal steroids

**Figure 1:**
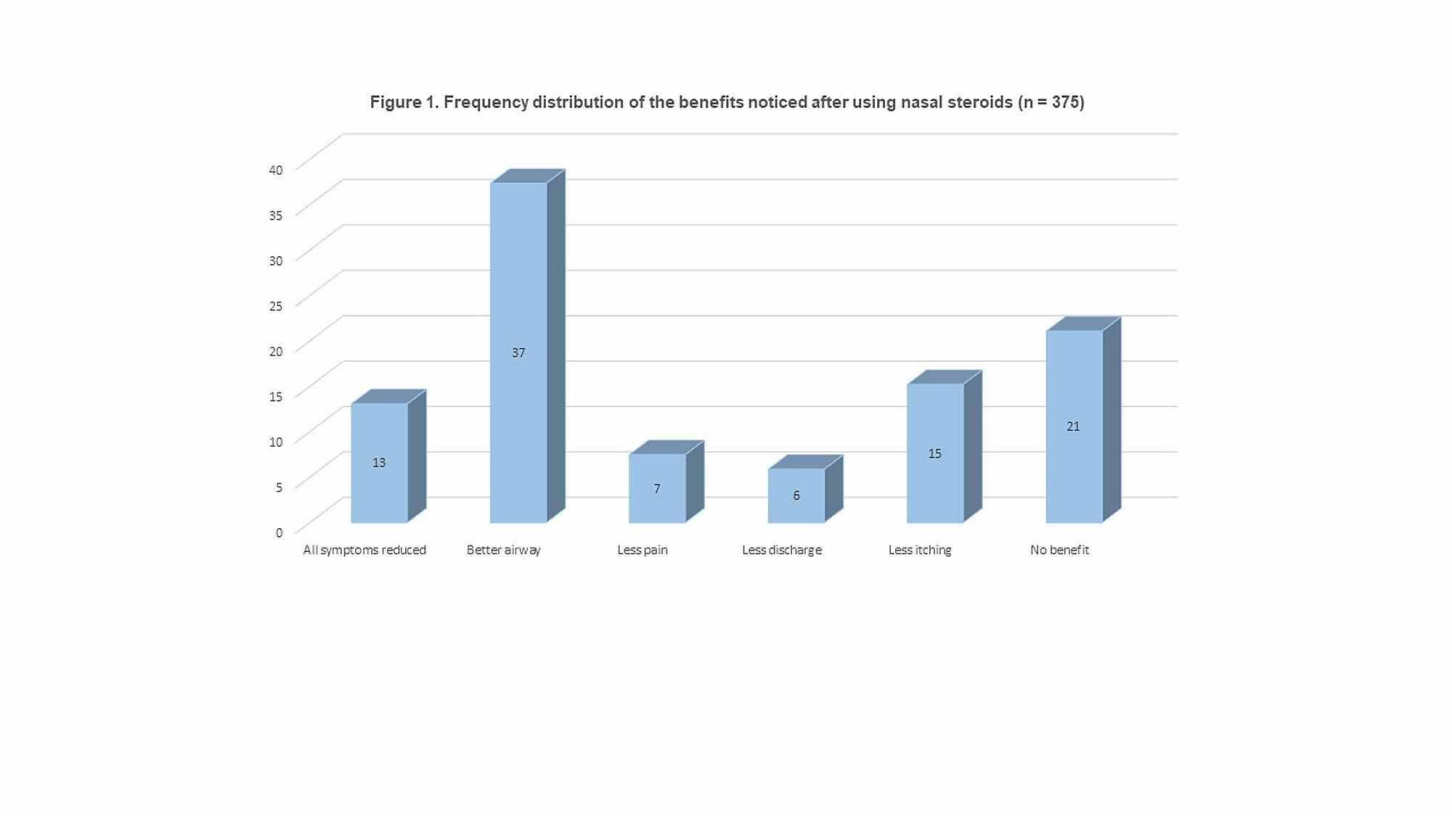
Frequency distribution of the benefits noticed after using nasal steroids (n = 375)

The participants reported that the benefits of using intranasal steroids were: better airway (37.3%, n = 140), less itching (15.2%, n = 57), less pain (7.5%, n = 28), and less nasal discharge (5.9%, n = 22). However, 21.1% (n = 79) thought that using intranasal steroids was not beneficial and only 13.1% (n = 49) thought they might reduce all symptoms (Figure 1).

**Figure 2:**
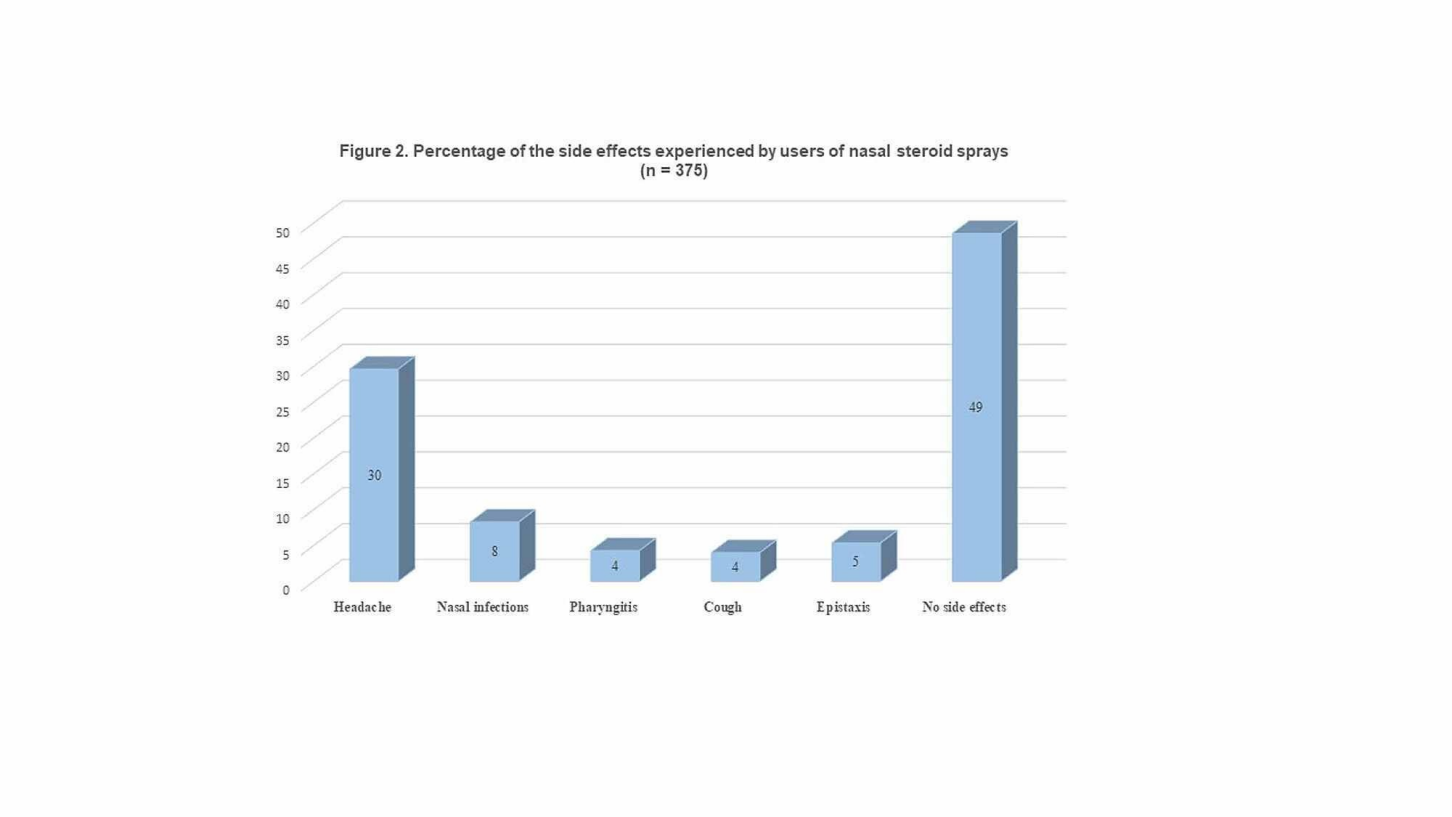
Percentage of the side effects experienced by users of nasal steroid sprays (n = 375)

 Lastly, the most frequent side effects reported by the participants were headache (29.6%), nasal infections (8.3%), and epistaxis (5.3%); but, most of the participants (48.5%, n = 182) reported no side effects (Figure 2). 

## Discussion

INCSs are one of the most important therapeutic options; they are considered to be the first-line treatment for patients with moderate-to-severe AR according to current published guidelines [24,25]. Patients’ adherence to the prescribed medications in their treatment plan is crucial for achieving maximum outcomes and optimal symptom relief. However, patients’ irrational fears and concerns about potential side effects could lead to discontinuing medical treatment, ultimately resulting in an unnecessary financial burden on individuals and affecting their quality of life [22,23]. Therefore, patients’ knowledge about and awareness of nasal steroid safety is important for establishing appropriate expectations and identifying preferences for therapy.

In our study, we found that the majority of the patients had used steroids before. However, a considerable number of patients with AR did not have appropriate knowledge about INCSs; two-fifths of study participants stated that INCSs were effective and important medications, and only a few reported INCSs to be unsafe. In a previous study conducted in Turkey to evaluate patients’ perspectives on oral and nasal steroids, 19% of the participants identified INCSs as being effective and important drugs, whereas 36% considered them to be unsafe medications [7]. Furthermore, most of the participants were unable to recognize and differentiate between related and unrelated side effects regarding steroid usage. Similar findings were reported in the literature among an Australian population; that study found that 67% of the patients had little or no knowledge about INCSs [4]. More than half of our patients had not received appropriate and correct advice during physician consultation visits, which can be an explanation for their poor response. This could have a serious impact on patients' health, delaying the ability to properly change their therapeutic regimen to obtain better results.

In the present study, the majority of the patients were compliant with the therapy regardless of their uncertainty about the medication’s safety, and they stated that they would use INCSs if prescribed without any objections. This may be explained by the fact that most of them perceived a benefit from using the medication, which would also explain their compliance. Another study conducted in the United States concluded that the patients had a clear preference for the sensory attributes of INCSs [19]. However, more than half of our participants had discontinued taking the medication once because they were concerned about its safety, did not want to experience unwanted side effects, or had difficulty using the medication. Moreover, half of the participants were concerned about using a nasal steroid but only half of them discontinued using it as a result. Similarly, a previous study conducted in multiple Middle East countries reported that 14% of the patients stopped using INCSs because of safety concerns, whereas 26% stopped because they experienced bothersome side effects [26]. Therefore, health care professionals play an important role in educating patients about these medications and addressing these issues during clinic visits. In our study, while most of the participants noted that their physicians had explained how to use steroids, a remarkable number of the participants (46.1%) did not recognize that a special technique should be followed to obtain maximum results. Physicians are encouraged to take more time and effort to provide medication-related information to their patients and demonstrate the correct technique of using prescribed nasal sprays to avoid side effects and unnecessary changes in treatment modality.

In our result, the patient's age was significantly correlated with perceptions of and adherence to using INCSs. Moreover, more males than females had discontinued the medication. Similar age and gender differences were reported in the literature, but the differences were not significant [27]. Moreover, among an older population, it was reported that subtle symptoms were the reason for noncompliance [4]. Furthermore, nonsmokers were more knowledgeable in terms of awareness of the special techniques for using nasal sprays. This can be explained by the desire of nonsmokers to be symptom-free, while long-term smokers might have concurrent comorbidities and are less likely to be attentive to such techniques. Furthermore, nonsmokers have experienced more benefits of using INCSs. Education level is another important factor; patients’ perception and practice of using INCSs were found to be significantly correlated with education. These findings are consistent with those reported in a study on a Turkish population, which stated that patients with higher education levels had a more positive attitude [27].

Limitations and strengths of the study

This study has several limitations. First, a study using a cross-sectional design can only suggest correlations, it cannot demonstrate causation. Moreover, the data were collected from a single-center, which may limit the generalizability of our findings. Therefore, we believe that further multicenter studies are needed to obtain more valid outcomes related to this issue. However, despite these limitations, this is the first study to shed light on patients’ concerns regarding the safety of using an intranasal steroid spray in the Saudi community.

## Conclusions

We conclude that addressing patients’ misconceptions about and concerns regarding the side effects of medications is a crucial step in managing many chronic diseases, and health care providers must take this into consideration. According to current guidelines, INCSs are first-line treatment for patients with moderate-to-severe AR. However, most of the participants in our study have safety concerns about using INCSs, which led to the discontinuation of the medication. One of the most significant findings to emerge from this study is that the patients’ age, gender, socioeconomic status, education level, and smoking status are potential barriers to facilitating positive perceptions of and adherence to their treatment management plan. Lastly, we emphasize the need for corrective measures to ensure better health outcomes. These important steps include raising the awareness of these concerns among health care providers and encouraging physicians to invest more time and effort in correcting these misconceptions to ensure patient adherence to medications and to avoid unnecessary changes in the treatment management plan.
